# Enzyme kinetics and distinct modulation of the protein kinase N family of kinases by lipid activators and small molecule inhibitors

**DOI:** 10.1042/BSR20140010

**Published:** 2014-03-18

**Authors:** Matthew D. Falk, Wei Liu, Ben Bolaños, Keziban Unsal-Kacmaz, Anke Klippel, Stephan Grant, Alexei Brooun, Sergei Timofeevski

**Affiliations:** *Pfizer Oncology Research Unit, Pfizer Inc., San Diego, CA 92121, U.S.A.; †Pfizer Worldwide Medicinal Chemistry, Pfizer Inc., San Diego, CA 92121, U.S.A.; ‡Pfizer Oncology Research Unit, Pfizer Inc., Pearl River, NY 10965, U.S.A.

**Keywords:** AGC kinase, cancer, kinase inhibitor, kinetic mechanism, lipid, protein kinase N (PKN), CaM, calmodulin, Cdk1/2, cyclin-dependent kinase 1/2, DAG, 1,2-dioctanoyl-sn-glycerol, DTT, dithiothreitol, HEK-293 cells, human embryonic kidney 293 cells, IP3, D-myo-inositol-1,3,5-triphosphate, MAPK, mitogen-activated protein kinase, MS/MS, tandem MS, PDK1, phosphoinositide-dependent kinase 1, PIF, PDK1-interacting fragment, PIP2, phosphatidylinositol-4,5-bisphosphate, PIP3, phosphatidylinositol-3,4,5-triphosphate, PKA, protein kinase A, PKC, protein kinase C, PKG, protein kinase G, PKN, protein kinase N, ROCK, Rho-associated kinase, S6K, S6 kinase

## Abstract

The PKN (protein kinase N) family of Ser/Thr protein kinases regulates a diverse set of cellular functions, such as cell migration and cytoskeletal organization. Inhibition of tumour PKN activity has been explored as an oncology therapeutic approach, with a PKN3-targeted RNAi (RNA interference)-derived therapeutic agent in Phase I clinical trials. To better understand this important family of kinases, we performed detailed enzymatic characterization, determining the kinetic mechanism and lipid sensitivity of each PKN isoform using full-length enzymes and synthetic peptide substrate. Steady-state kinetic analysis revealed that PKN1–3 follows a sequential ordered Bi–Bi kinetic mechanism, where peptide substrate binding is preceded by ATP binding. This kinetic mechanism was confirmed by additional kinetic studies for product inhibition and affinity of small molecule inhibitors. The known lipid effector, arachidonic acid, increased the catalytic efficiency of each isoform, mainly through an increase in *k*_cat_ for PKN1 and PKN2, and a decrease in peptide *K*_M_ for PKN3. In addition, a number of PKN inhibitors with various degrees of isoform selectivity, including potent (*K*_i_<10 nM) and selective PKN3 inhibitors, were identified by testing commercial libraries of small molecule kinase inhibitors. This study provides a kinetic framework and useful chemical probes for understanding PKN biology and the discovery of isoform-selective PKN-targeted inhibitors.

## Abstract 2

We conducted kinetic analysis of the relatively unexplored PKN family and effects of lipids, and identified potent inhibitors with various isoform selectivity. The kinetic mechanism, lipid activators and inhibitors could be useful for understanding PKN biology and developing PKN-targeted therapies.

## INTRODUCTION

The PKN (protein kinase N) family, also known as the PRKs (protein kinase C-related kinases), belongs to the AGC family of kinases and consists of three members: PKN1/PRK1, PKN2/PRK2 and PKN3. The PKN enzymes are ~100 kDa proteins and are closely related to the novel isoforms of the protein kinase C enzyme superfamily. Similar to other AGC kinases, PKN1–3 are structurally conserved in the activation loop, as well as C-terminal segments termed the hydrophobic and turn motif. PKN1–3 requires phosphorylation of the activation loop by PDK1 (phosphoinositide-dependent kinase 1) as well as turn motif phosphorylation for full catalytic activity [1]. An activation mechanism has been reported for PKN2 [2], whereby hydrophobic-motif interaction with the PIF (PDK1-interacting fragment)-pocket of PDK1 allows for activation loop phosphorylation, and upon turn motif phosphorylation, the C-terminal domain of PKN2 dissociates from PDK1 and interacts with its own catalytic domain, stabilizing an active conformation. This activation mechanism is similar for S6K (S6 kinase) and SGK (serum/glucocorticoid regulated kinase), and differs slightly for AKT, which lacks a requirement for association with the PIF-pocket of PDK1 [3]. While the primary amino acid sequence of the kinase domains of PKN2 and PKN3 are 87% and 70% conserved with PKN1, respectively, they differ to a large extent in their N-terminal regions which have been shown to interact with Rho- and Rac-family GTPases [1,4,5].

Similar to the novel family of PKCs (protein kinase Cs; PKCδ, PKCε, PKCθ, PKCη), PKN1–3 contain a Ca^2+^-independent novel C2-domain between the N-terminal and kinase domains [6]. While PKCθ also contains the lipid binding C1A and C1B domains common to other PKC isoforms, a model was proposed whereby the C2-domain of PKCθ binds the lipid-rich plasma membrane and unleashes the protein from a folded state, leading to a fully active enzyme [7]. The C2-domains of other novel PKC members have been characterized, and have been revealed to be important for membrane targeting and enzymatic activation, as well as protein–protein interactions [8–10]. While the function of the C2-domain is not yet clear for the PKNs, it too may be involved in lipid binding and activation. Stimulation of PKN kinase activity in the presence of lipids *in vitro* is well documented [1,11–13], and PKN1 has been shown to signal through RhoA at the plasma membrane [14]. In a seminal study to determine the structural basis for lipid activation of PKN1 activity, Yoshinaga et al. [15] made a series of truncation mutants and found that while full-length PKN1 displayed low basal activity and demonstrated a dependence on arachidonic acid, N-terminal truncation at residue 511 markedly increased specific activity and decreased arachidonic acid sensitivity. A peptide corresponding to residues 455–511 inhibited PKN1 activity in a dose-dependent manner and was two-orders of magnitude less potent in the presence of arachidonic acid. It was proposed that residues 455–511 composed an autoinhibitory domain within PKN1 that is released in the presence of lipids. This work addressed a hypothesis that interaction of lipids with the PKNs may free the protein from a compact, inhibited state, leading to enzymatic activation and downstream signalling, similar to the PKC family of kinases.

Individual PKN isoforms vary in tissue distribution, with PKN1 and PKN2 ubiquitously expressed, and PKN3 mainly restricted to various tumour tissues [1,16]. As downstream effectors of Rho- and Rac GTPases, PKNs are implicated in a variety of normal physiological process, such as cytoskeletal remodelling and cell cycle progression, as well as oncogenic processes [16–22]. As such, the PKNs have begun to be scrutinized as possible drug targets for the treatment of cancer. PKN1 has been linked to prostate cancer through its interaction with the androgen receptor [23,24]. PKN2 was recently implicated in triple negative breast cancer [25], and PKN3 was found to be required for malignant growth in a prostate tumour model downstream of an activated PI3K (phosphoinositide 3-kinase) [16,22] and is targeted using an RNAi (RNA interference) approach for solid tumours in Phase I clinical trials [26]. With the interest in PKN-targeted agents growing, a further understanding of PKN enzymatic regulation is required. Recombinant PKN1 [27,28], PKN2 [28–30] and PKN3 [27,28] have been used in prior works, but no detailed enzyme kinetics have been reported, and effects of lipids have not been directly compared for all three isoforms. In addition, there were very few reports of small molecule inhibitors for PKN1 or PKN2, and none for PKN3, to our knowledge. To that extent, using recombinant full-length human enzymes and a synthetic peptide substrate, we determined the kinetic mechanism of PKN isoforms. To deduce how the function of PKN1–3 may be regulated, we have investigated differential lipid sensitivities of all three isoforms and determined the effects of arachidonic acid on the enzyme catalytic parameters. In addition, through compound library screening, we sought to exploit the minimal differences in the ATP binding sites of PKN1–3, and have identified potent small molecule inhibitors with varying degrees of isoform selectivity, potentially useful as tool compounds to dissect PKN-dependent biology.

## EXPERIMENTAL

### Materials

Microtitre 96-well polypropylene plates and 384-well non-binding, low volume plates were purchased from Corning Life Sciences. PKN substrate peptide (5FAM-Ahx-GGGGPKGPGRRGRRRTSSFAEGG-COOH, where Ahx is an aminohexane linker) and PKN3-PRL inhibitor peptide (NH2-PRLQRQERIFSKRRG-COOH) were synthesized and purified to at least 95% purity by CPC Scientific. CHAPS detergent was purchased from Pierce. Arachidonic acid was purchased from Cayman Chemical Company. All other lipids were purchased from Avanti Polar Lipids. Phospho-PRK1 (Thr^774^)/PRK2 (Thr^816^) antibody, which has been found to cross-react with PKN3 [16], was purchased from Cell Signaling Technology. Y27632 (CAS No. 146986-50-7) was synthesized by Pfizer, and is also available from Sigma-Aldrich. Kinase inhibitor libraries were acquired from Biomol/Enzo Life Sciences and EMD Calbiochem/Millipore, and were used for testing with PKN1, PKN2 and PKN3 at single dose followed up by a dose-response (*K*_i_) for representative ‘hits’. JAK3 Inhibitor VI (CAS No. 856436-16-3), PKR Inhibitor (CAS No. 608512-97-6) and Cdk1/2 (cyclin-dependent kinase 1/2) inhibitor III (CAS No. 443798-55-8) were from the EMD Calbiochem/Millipore Kinase Inhibitor Libraries. GF 109203X (CAS No. 133052-90-1), H-8 (CAS No. 84478-11-5), HA-1077 (CAS No. 103745-39-7), KN-62 (CAS No. 127191-97-3), PKC-412 (CAS No. 120685-11-2), PP1 (CAS No. 172889-26-8), Ro 31-8220 (CAS No. 138489-18-6), SB-202190 (CAS No. 152121-30-7) were from the Biomol kinase inhibitor library.

LabChip EZ Reader 12-sipper chip, CR3 and CR8 coating reagent were purchased from Caliper Life Sciences/PerkinElmer. Anti-FLAG M2 antibody, DTT (dithiothreitol), ATP, ADP, MgCl_2_, Brij-35, EDTA and all other reagents were purchased from Sigma-Aldrich. All proteins used in the biochemical studies were produced by Pfizer.

### Expression and purification of full-length human PKN1, PKN2 and PKN3

PKN1 and PKN2 genes were optimized for mammalian expression and synthesized at GenScript, while native sequence derived from cDNA was used for PKN3. The sequence encoding FLAG tag (DYKDDDDK) was fused to the N-terminus of each gene and then cloned into the pcDNA4/TO vector (Invitrogen). Human PKN1 and PKN2 were transfected into 300 ml of HEK-293 cells (human embryonic kidney 293 cells) using Freestyle Max transfection reagent (Invitrogen) according to the manufacturer's protocol, and cells were harvested 48 h after transfection. Due to low expression level of PKN3 in the transient transfection format, HEK-293-TRex PKN3 stable cells were generated. Flag-PKN3 cDNA was cloned into the pcDNA4/TO vector as described before [22], and this construct was transfected into the HEK-293-TRex cell line (Invitrogen) which stably express the Tet repressor. After 48 h, transfected cells were selected for resistance to zeocin (300 μg/ml). Cell clones appeared after 10 to 15 days. Isogenic pooled clones were expanded and used for induction of Flag-PKN3 by doxycycline (Sigma–Aldrich). These cells were adopted into suspension in FreeStyle 293 medium (Invitrogen) containing 10% FBS (fetal bovine serum), 50 μg/ml G418, 300 μg/ml zeocin and 1% penicillin/streptomycin. Cells were cultured in shake flasks and diluted to 0.5×10^6^/ml at day 0, induced with 1 μg/ml of doxycycline on day 1 and harvested 48 h after induction.

Frozen cells were lysed with 5 volumes of lysis buffer (30 mM Tris, pH 7.5, 150 mM NaCl, 10% glycerol, 1% Triton X-100, 25 mM beta-glycerol phosphate, 50 mM sodium flouride, 10 mM sodium pyrophosphate, 0.5 mM DTT and Roche Complete Protease Inhibitor Cocktail (Roche Applied Science), and purification and elution of the Flag tagged PKNs was performed using anti-FLAG M2 Affinity Gel and 3x-FLAG peptide using standard protocols. Purified full-length PKN proteins were further analysed for homogeneity using analytical size exclusion chromatography and their phosphorylation state was confirmed by MS and Western-blot analysis.

*PKN MS analysis–* To determine the phosphorylation state of specific amino acids, PKN1–3 were subjected to mass spectrometric analysis. Recombinant PKN protein was diluted in 50 mM ammonium bicarbonate to a concentration of 50 ng/μl, and heat denatured at 90°C for 15 min. Protein was reduced with 5 mM DTT at 37°C for 1 h, and alkylated with 10 mM iodoacetamide at 25°C in the dark for 1 h. Trypsin Gold (Promega) was added to the samples at a ratio of 1:20 (w/w), and incubated for 16 h at 37°C. The resulting PKN peptides were then analysed on a LTQ mass spectrometer (Thermo Fisher Scientific) coupled to a Proxeon nanoLC. PKN digest sample was injected on to a Reprosil ProteCol Trap C18-AQ (SGE Analytical Sciences), where it was desalted, then eluted on to a Halo ES-C18 column (Michrom BioResources), 0.2×150 mm, for analytical separation at a flow rate of 1.8 μl/min. A 50 min gradient was employed, which consisted of a 35 min gradient from 2–40% acetonitrile with 0.1% (v/v) formic acid, after which, the acetonitrile increased to a maximum 80% for the remainder of the run. MS data were collected in ‘triple-play’ mode, which uses a full scan at 400–2000 *m*/*z* for selection of the three most predominant eluting peptides, followed by a high resolution zoom scan, and MS/MS (tandem MS) of the respective isolated peak of interest. MS/MS spectra were collected with an isolation window of 3 *m*/*z* and collision activation energy (CAD) of 35 eV. MS/MS data were then processed using Agilent Spectrum Mill 4.0 software. The sequence of PKN1–3 was appended to the database, and peptides were searched directly against that protein sequence. Fixed modification of carbamidomethylation, along with variable methionine oxidation and phosphorylation (serine, threonine and tyrosine) were included in the sequence search. Precursor mass tolerance was set at 1.0 Da, and 0.7 Da for the fragment ion. Peptides were validated by fixed thresholds of forward–reverse scores >8 and scored peak intensities >70. The search parameters were set for up to three missed cleavage sites.

### Lipid preparation

Lipids were supplied as a solid powders, ethanol, or chloroform solutions. All solids were dissolved directly into assay buffer and briefly water bath sonicated at room temperature for 10 min. Lipids supplied as solutions in organic solvent were dried under a gentle stream of nitrogen, resuspended in assay buffer, and similarly sonicated. All lipids were prepared fresh daily.

### PKN kinase assay

The activity of all PKN proteins was monitored through the use of a highly sensitive off-chip mobility shift assay utilizing a LabChip EZ Reader II instrument (Caliper Life Sciences/PerkinElmer); this assay format has been described elsewhere [31]. Kinase activity was monitored through the phosphorylation of a 5-FAM-labelled PKN substrate peptide found through Pfizer in-house optimization efforts and derived from the amino acid sequence of GSK3α (accession no. AAH51865.1) with the following sequence: 5-FAM-C6-GGGGPKGPGRRGRRRTSSFAEGG-NH2. The likely site of phosphorylation is underlined, as determined through comparison with the published phosphorylation consensus sequence for PKN1 and PKN3 substrates [27]. Reaction buffer contained 25 mM Tris, pH 8.0, 5 mM MgCl_2_, 0.002% CHAPS and 2 mM DTT, with indicated protein, ATP and peptide concentrations. Peptide concentration was adjusted according to purity and peptide content. For inhibition studies, the inhibitor was pre-incubated with enzyme for 15 min at room temperature. All reactions were initiated with ATP and conducted at room temperature (~22°C). Reactions were terminated by the addition of EDTA, pH 8.0, at a final concentration of 25 mM. All reaction rates were determined to be linear with respect to time and enzyme concentration where no more than 20% of peptide substrate had been converted to product. Aliquots of each reaction were transferred in duplicate to a 384-well plate and analysed on a LabChip EZ Reader II with separation buffer containing 100 mM Hepes, pH 7.5, 0.015% Brij-35, 1 mM EDTA, 0.1% CR3 coating reagent, 1×CR8 coating reagent and 5% DMSO, with the following settings: downstream voltage: −500 V, upstream voltage: −2300 V, base pressure: −0.5 psi; screen pressure: −1.7 psi.

### Enzyme kinetics and inhibition analysis

All data were globally fit using non-linear regression and GraphPad Prism software. Non-linear data were converted to double-reciprocal Lineweaver–Burk plots for visual and presentation purposes only. Kinetic data were analysed in duplicate, and each plot is representative of at least two independent experiments. Equations (1)–(4) used for kinetic analysis were described previously [32,33].

To determine the kinetic parameters of a steady-state ordered Bi–Bi reaction mechanism, data were fit to eqn (1):

(1) $$\begin{array}{c} v=\frac{V_{\max}\left[\mathrm{ATP}\right]\left[\mathrm{Pep}\right]}{\left(K_{\mathrm{ia}}\right)\left(K_{M,\mathrm{Pep}}\right)+\left(K_{M,\mathrm{Pep}}\right)\left[\mathrm{ATP}\right]+\left(K_{M,\mathrm{ATP}}\right)\left[\mathrm{Pep}\right]+\left[\mathrm{ATP}\right]\left[\mathrm{Pep}\right]} \end{array}$$

For enzyme inhibition studies, data were fit to eqns (2)–(4) for competitive, non-competitive and uncompetitive inhibition, respectively:

(2) $$v=\frac{V_{\max}\left[S\right]}{\left[S\right]+K_{M}\left(1+\frac{\left[I\right]}{K_{i}}\right)}$$

(3) $$v=\frac{V_{\max}\left[S\right]}{\left(\left[S\right]+K_{M}\right)\left(1+\frac{\left[I\right]}{K_{i}}\right)}$$

(4) $$v=\frac{V_{\max}\left[S\right]}{\left[S\right]\left(1+\frac{\left[I\right]}{\alpha K_{i}}+K_{M}\right)}$$

For eqns (1)–(4), *v* is measured velocity, *V*_max_ is maximum velocity, [ATP], [Pep] and [*S*] are concentrations of ATP, peptide and varied substrate, respectively; *K*_ia_ is the equilibrium dissociation constant of ATP, *K*_M,ATP_, *K*_M,Pep_ and *K*_M_ are the Michaelis–Menten constants for ATP, peptide and the varied substrate, respectively. [*I*] is the inhibitor concentration, α describes the preference for inhibitor binding to either free or substrate bound enzyme and *K*_i_ is the inhibitor equilibrium dissociation constant.

*K*_i_ values for representative inhibitors were determined by fitting initial reaction velocities to eqn (2) for competitive inhibition using the method of non-linear least-squares using GraphPad Prism. Turnover number (*k*_cat_) was calculated by normalizing *V*_max_ values for enzyme concentration. To obtain lipid EC_50_ values, as well as fold rate-activation, data were fit to eqn (5):

(5) $$v=v_{0}+\frac{\left(v_{i}-v_{0}\right)}{1+10^{\left(log\mathrm{EC}50-X)(\mathrm{Hill}\;\mathrm{Slope}\right)}}$$

where *v* is measured velocity, *v*_o_ and *v*_i_ are the corresponding plateaus at low and high lipid concentrations, respectively; EC_50_ is the concentration at which enzyme activity is stimulated by 50%, and *X* is lipid concentration in log units.

## RESULTS

### PKN activation loop and turn motif phosphorylation analysis

**Scheme 1:**
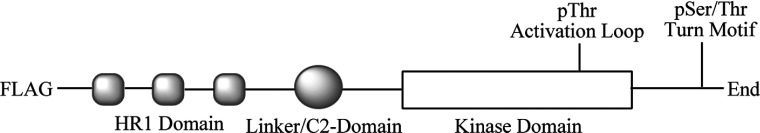
Domain organization and sites of phosphorylation for recombinant full-length PKN1–3 used in this study

**Figure 1:**
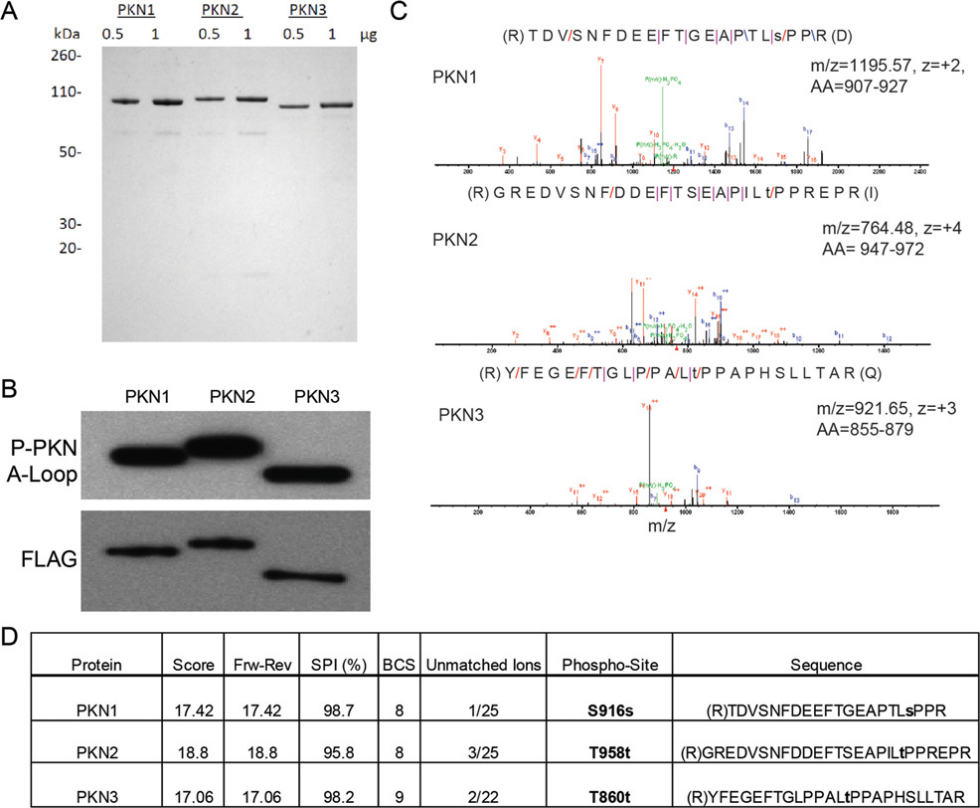
Characterization of full-length PKN enzymes (**A**) SDS/PAGE: FLAG-tagged, full-length PKN1, PKN2 and PKN3 were expressed in HEK-293 cells and purified as described in the Experimental section. 0.5 μg and 1 μg of protein was separated by SDS/PAGE and stained with Simply Blue (Invitrogen). (**B**) Western blotting: 20 ng PKN1–3 were separated by SDS/PAGE, transferred to nitrocellulose, and probed with the indicated antibodies. (**C**) MS/MS spectra of the turn motif peptide for PKN1, PKN2 and PKN3, respectively. (**D**) MS/MS scoring of PKN1–3 turn motif peptide provides localization of phosphorylation site, performed as described under the Experimental section.

Full-length, FLAG-tagged human PKN1, PKN2 and PKN3 were expressed in mammalian HEK-293 cells (summary of protein construct used in Scheme 1). All proteins were purified to greater than 85% as determined by SDS/PAGE and Coomassie Blue staining (Figure 1A). The phosphorylation of the activation loop of PKN1–3 was confirmed by Western blotting (Figure 1B). Incubation of PKN proteins with recombinant PDK1 in the presence of Mg-ATP had no effect on further phosphorylation of the activation loop, suggesting that the latter was already fully phosphorylated (results not shown). An LC (liquid chromatography)-MS/MS approach was employed specifically to probe the phosphorylation state of the turn motif due to lack of commercial antibodies. MS/MS data of the tryptic digest provided varied sequence coverage of the three isoforms: PKN1 (53%), PKN2 (51%) and PKN3 (31%). From MS/MS data, identification of potential sites of phosphorylation (tyrosine, serine and threonine) indicated only the peptide containing the turn motif residue as a high-confidence phosphosite. This turn motif peptide was consistently observed fully phosphorylated indicating high occupancy. A baseline chromatograph is shown for the PKN2 tryptic digest, as an example, in Figure S1 (available online at http://www.bioscirep.org/bsr/034/bsr034e097add.htm), as well as the associated extracted ion chromatogram for the respective phosphorylated turn motif peptide (amino acids 947–972). The MS/MS data derived from the resulting fragmentation peptides for PKN1, PKN2 and PKN3 (Figure 1C) were evaluated in Agilent Spectrum Mill using a variety of criteria (Figure 1D). The phosphopeptides listed in Figure 1(D) were the respective top ranking hits to localize the site of phosphorylation in the turn motif. Previous reports had suggested that PKN isoforms are able to autophosphorylate *in vitro*, leading to an increase in activity [1,34,35]. Incubation of full-length protein with Mg-ATP for up to 3 h led to only a marginal increase in initial rates for PKN1, PKN2 and PKN3 kinase reactions (results not shown). With the degrees of activation relatively small, proteins not subjected to pre-incubation with Mg-ATP was used throughout this study.

### Response to lipid activators

**Figure 2:**
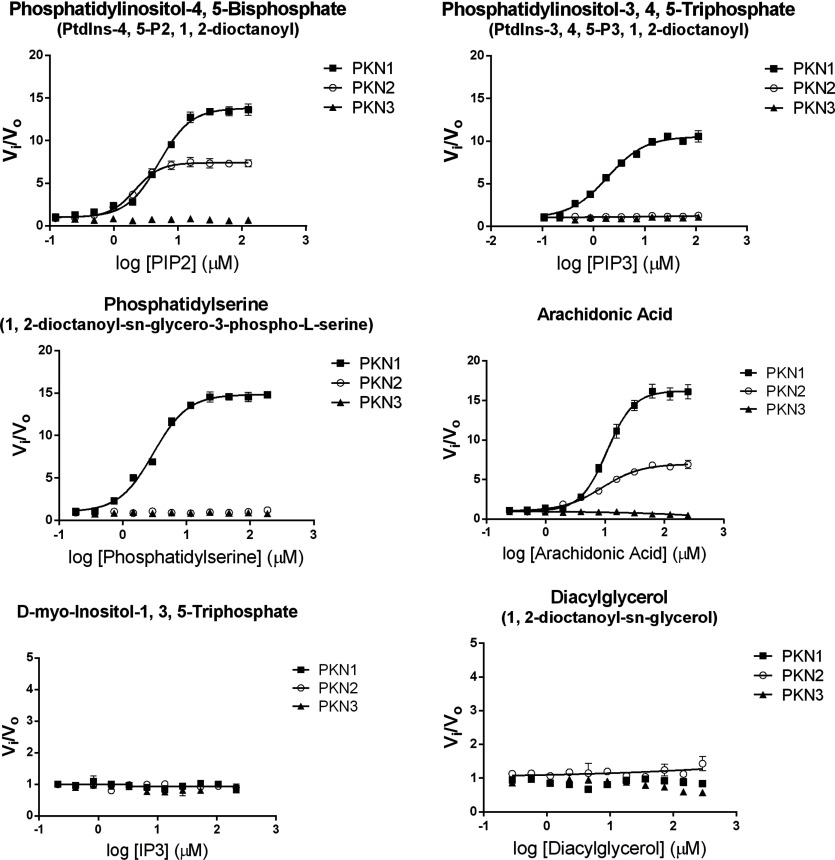
PKN responses to lipid activators in biochemical assays Lipids were prepared as described under the Experimental section, and kinase activity of PKN isoforms was measured using off-chip mobility shift assay described in the Methods section. Each lipid was titrated in the presence of 1 nM, 5 nM and 3 nM PKN1–3, respectively, 3 μM peptide and 500 μM ATP. *v*_i_ is the initial rate at each concentration of lipid activator and *v*_o_ is the initial rate in the absence of lipid.

The kinase activity of the PKN family has been reported to be responsive to fatty acids, such as arachidonic and linoleic acid as well as phosphatidylinositols [1,28,36]. To determine the effects that lipids may have on the kinetics of the individual PKN isoforms, we determined an EC_50_ value and maximal increase in initial rates under saturating ATP conditions of a small subset of potentially relevant signalling lipids against each PKN isoform using GSK3α derived peptide substrate (Figure 2 and Table 1). PKN1 initial rates were similarly increased by PIP2 (phosphatidylinositol-4,5-bisphosphate), PIP3 (phosphatidylinositol-3,4,5-triphosphate), phosphatidylserine and arachidonic acid (approximately 10–16-fold). Similar to PKN1, PKN2 kinase activity was increased in the presence of PIP2 and arachidonic acid. However, PKN2 kinase activity was not affected by either phosphatidylserine or PIP3. The activation profile of PKN3 by lipids differed greatly from PKN1 and PKN2. In contrast, stimulation of PKN3 catalytic activity was not observed with any of the lipids tested. However, on closer examination, arachidonic acid produced complex effects on PKN3. Kinase activity of PKN3 was slightly stimulated at a relatively low (5 μM) concentration of arachidonic acid from the kinetic analysis below, but was significantly inhibited at higher concentrations of this lipid (Figure 2). Arachidonic acid stimulation of PKN1 and PKN2 kinase activity was reported by others [28,34,35]. In this work, we observed pronounced differences in stimulation of the PKN isoforms by archidonic acid, and conducted detailed kinetic analysis of the PKN family proteins in the presence and absence of arachidonic acid.

**Table 1:** Summary of effects of lipid activators on kinase activity of PKN isoforms Lipids were prepared as described under the “Experimental” section. Kinase activity was measured with saturating ATP using the off-chip mobility shift assay, and EC_50_ values and maximal rate were obtained by fitting data to Eqn (5), as described under the “Experimental” section. EC_50_ was not determined (ND) for lipids that caused less than 2-fold maximal activation.

	PKN1		PKN2		PKN3	
Lipid	EC_50_ (μM)	Maximal fold activation*	EC_50_ (μM)	Maximal fold activation*	EC_50_ (μM)	Maximal fold activation*
PIP2	5±1	14	2±1	7	ND	<2
PIP3	2±1	11	ND	<2	ND	<2
Phosphatidylserine	3±1	15	ND	<2	ND	<2
Arachidonic Acid	11±1	16	9±1	7	ND	<2
IP3	ND	<2	ND	<2	ND	<2
DAG	ND	<2	ND	<2	ND	<2

*Maximal activation depends on substrate concentrations and applies only for the described conditions.

### Steady-state kinetic analysis

**Figure 3:**
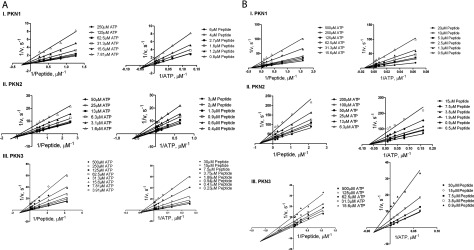
Bisubstrate kinetics of PKN isoforms (**A**) Bisubstrate kinetic analysis of PKN1, PKN2 and PKN3 in the presence of arachidonic acid. Lineweaver–Burk plots of 1/*v* versus 1/[peptide] (left) and 1/*v* versus 1/[ATP] (right) using off-chip mobility shift assay, described under the Experimental section. (I) Initial velocities for PKN1 were measured in the presence of 7.8–250 μM ATP, 0.8–6 μM peptide, 100 μM arachidonic acid and 0.5 nM enzyme. (II) Initial velocities for PKN2 were measured in the presence of 1.6–50 μM ATP, 0.4–3 μM peptide, 100 μM arachidonic acid and 2 nM enzyme. (III) initial velocities for PKN3 were measured in the presence of 3.9–500 μM ATP, 0.2–30 μM peptide, 5 μM arachidonic acid and 2 nM enzyme. (**B)** Bisubstrate kinetic analysis of PKN1, PKN2 and PKN3 in the absence of arachidonic acid. Lineweaver–Burk plots of 1/*v* versus 1/[peptide] (left) and 1/*v* versus 1/[ATP] (right) using off-chip mobility shift assay, described in the Experimental section. (I) Initial velocities for PKN1 were measured in the presence of 15.6–500 μM ATP, 0.6–20 μM peptide and 5 nM enzyme. (II) Initial velocities for PKN2 were measured in the presence of 6.3–200 μM ATP, 0.5–15 μM peptide and 20 nM enzyme. (III) Initial velocities for PKN3 were measured in the presence of 15.6–500 μM ATP, 0.9–30 μM peptide and 4 nM enzyme.

**Figure 4:**
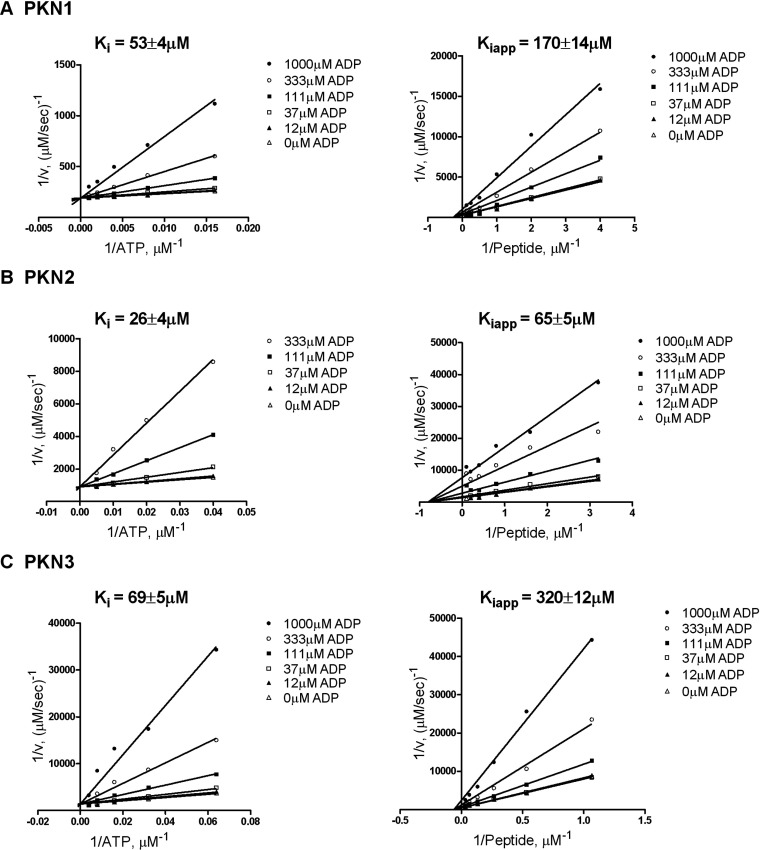
Product inhibition studies of PKN1, PKN2 and PKN3 in the presence of ADP Lineweaver–Burk plots of 1/*v* versus 1/[ATP] (left) and 1/*v* versus 1/[peptide] (right) with indicated fixed concentration of ADP, using off-chip mobility shift assay described in the Experimental section. (**A**) Initial velocities of PKN1 with 3 μM peptide and 31–1000 μM ATP (left); and with 50 μM ATP and 0.3–10 μM peptide (right), with 1 nM enzyme and 100 μM arachidonic acid. (**B**) Initial velocities of PKN2 with 2 μM peptide and 6–200 μM ATP (left); and with 20 μM ATP and 0.3–10 μM peptide (right), with 2 nM enzyme and 100 μM arachidonic acid. (**C**) Initial velocities of PKN3 with 5 μM peptide and 7.8–250 μM ATP (left); and 50 μM ATP and 0.9–30 μM peptide (right), with 4 nM enzyme and 5 μM arachidonic acid.

**Figure 5:**
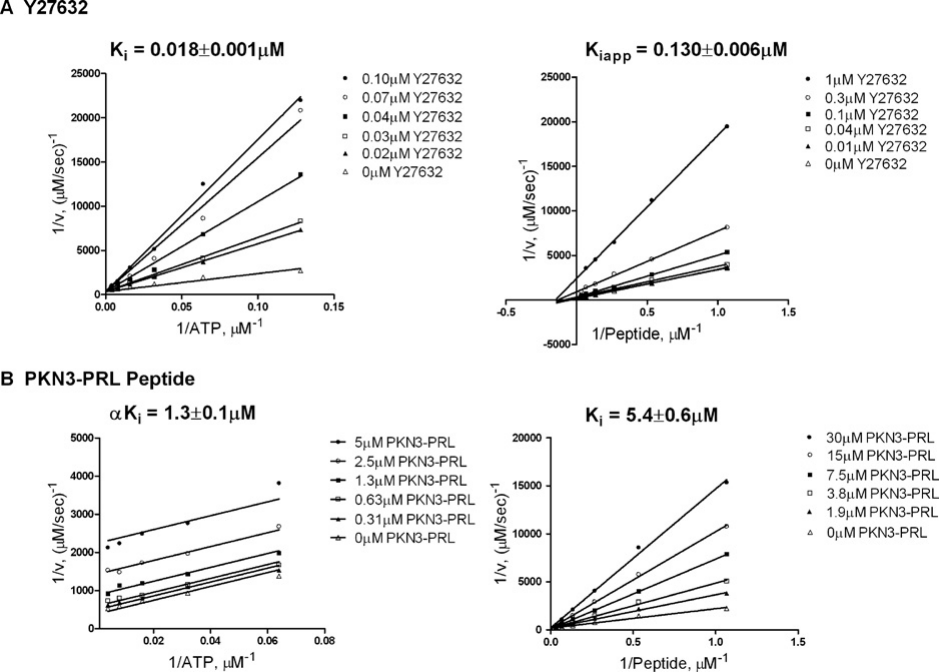
Phosphoacceptor substrate and ATP competitive inhibition of PKN3 Lineweaver–Burk plots of 1/*v* versus 1/[ATP] or 1/*v* versus 1/[peptide] for PKN3 in the presence of ATP or phosphoacceptor substrate-competitive inhibitors using off-chip mobility shift assay, described in the Experimental section. (A) Initial velocities of PKN3 with 6 μM peptide substrate and 6.3–200 μM ATP (left); and with 250 μM ATP and 0.94–30 μM peptide substrate (right), with indicated concentration of Y27632, 4 nM enzyme and 5 μM arachidonic acid. (B) Initial velocities of PKN3 with 6 μM peptide substrate and 7.8–250 μM ATP (left); and with 250 μM ATP and 0.9–30 μM peptide substrate (right), with indicated concentration of PKN3-PRL peptide inhibitor, 4 nM enzyme and 5 μM arachidonic acid.

To determine the catalytic contribution of lipid activation, as well as the kinetic mechanism and order of substrate addition during peptide phosphorylation, bisubstrate kinetic analysis was performed in the presence and absence of arachidonic acid by varying ATP and peptide substrate in a matrixed format. Since PKN1 and PKN2 achieved maximal initial velocity in the presence of 100 μM arachidonic acid, this concentration was chosen for these two enzymes. The effects of arachidonic acid at 5 μM (below the inhibitory concentration) on PKN3 kinetics were also examined. Transformation of initial velocity data to Lineweaver–Burk plots displayed lines intersecting to the left of the *y*-axis, consistent with a sequential mechanism, whereby a ternary complex forms between enzyme and substrates before product release (Figures 3A and 3B). To distinguish ordered versus random kinetic mechanism, product inhibition (using ADP, Figure 4) and substrate competitive inhibition studies (using Y27632 and PKN3-PRL peptide, Figure 5) were conducted. These data sets were consistent with an ordered Bi–Bi kinetic mechanism, as further explained below. The catalytic parameters generated through global fitting of the data to eqn (1), are presented in Table 2. There is considerable difference in kinetic constants between isoforms, both in the presence and absence of arachidonic acid. Arachidonic acid caused an 18-fold increase in *k*_cat_ for PKN1 and a 9-fold increase in *k*_cat_ for PKN2, which primarily contributed to the 32-fold and 12-fold increase in catalytic efficiency, respectively. Although arachidonic acid at 5 μM had no effect on *k*_cat_ for PKN3, it actually led to a 4-fold decrease in *K*_M,Pep_, and corresponding increase in the catalytic efficiency (*k*_cat_/*K*_M,Pep_). The ATP *K*_M_ for PKN1 was relatively unaffected by arachidonic acid, while ATP *K*_M_ for PKN2 and PKN3 was marginally reduced by 6- and 3-fold, respectively. As stated above, PKN1 was actually dramatically more sensitive to arachidonic acid with regard to processing the peptide substrate (as measured by *k*_cat_/*K*_M,Pep_). On the other hand, PKN2 actually showed the highest increase (45-fold) with regard to processing of ATP upon arachidonic acid stimulation (as measured by *k*_cat_/*K*_M,ATP_) (Table 2). We cannot discount the idea that a physiological protein substrate, rather than a synthetic peptide, or the addition of effector proteins, such as Rho or Rac, may have an influence on the catalytic properties of PKNs.

**Table 2:** Kinetic parameters of PKN1, PKN2 and PKN3 Means±S.D. (*n* ≥ 2) using off-chip mobility shift assay for full-length, FLAG-tagged PKN1, PKN2 and PKN3 purified from HEK-293 cells as described in the Experimental section.*

Kinase		*k*_cat_ (s^−1^)	*K*_M,ATP_ (mM)	*K*_ia_ (μM)	*k*_cat_*/K*_M,ATP_ (M^−1^ s^−1^)	*K*_M,Pep_ (μM)	*k*_ca*t*_*/K*_M,Pep_ (M^−1^ s^−1^)
PKN1	0 μM AA	0.7±0.1	0.24±0.02	230±10	3×10^3^	13±1	5×10^4^
	100 μM AA	13±6	0.18±0.05	19±10	7.2×10^4^	8±3	2×10^6^
PKN2	0 μM AA	0.10±0.01	0.06±0.02	50±8	2×10^3^	1.8±0.8	5.6×10^4^
	100 μM AA	0.9±0.1	0.010±0.003	2±1	9×10^4^	1.3±0.3	7×10^5^
PKN3	0 μM AA	1.0±0.1	0.22±0.03	10±9	5×10^3^	31±5	3×10^4^
	5 μM AA	1.1±0.1	0.067±0.005	14±3	1.6×10^4^	7.9±0.5	1.4×10^5^

*AA, arachidonic acid.

### Product inhibition by ADP

The pattern of intersecting lines generated from bisubstrate kinetic analysis eliminated the possibility of a ping-pong (double displacement) and rapid equilibrium ordered mechanism of peptide phosphorylation. Parallel lines or lines intersecting at the *y*-axis for the second added substrate would be produced for either of these mechanisms, respectively. To further discriminate between an ordered and random mechanism of substrate binding, ADP was used as a product inhibitor. Initial velocities were measured for PKN1, PKN2 and PKN3 in the presence of fixed concentrations of ADP while varying either ATP or peptide substrate. ADP inhibited all isoforms similarly, behaving as a competitive inhibitor with respect to ATP and a non-competitive inhibitor with respect to peptide substrate (Figure 4). This is consistent with an ordered Bi–Bi mechanism where ATP is the first substrate to bind. If the binding of substrates were random, non-competitive inhibition would have been seen varying both peptide and ATP under these conditions. Since all isoforms followed the same kinetic mechanism, PKN3, as the least characterized isoform, was chosen for further kinetic analysis.

### ATP- and peptide substrate-competitive inhibition

**Scheme 2:**
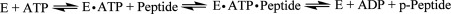
Reaction scheme depicting the steady-state ordered Bi–Bi mechanism of PKN1–3

Two inhibitors were chosen to further confirm the order of substrate binding with respect to PKN3. Y27632 is a known inhibitor of PKN1 and PKN2 [28,37], as well as other kinases [29]. In addition, a peptide, designated PKN3-PRL, was generated corresponding to a putative autoinhibitory domain in PKN3. This autoinhibitory domain and the corresponding peptide were initially described for PKN1 [15,38]. Initial velocity measurements varying either ATP or peptide substrate in the presence of Y27632 revealed competitive inhibition with respect to ATP, and non-competitive inhibition with respect to peptide substrate (Figure 5A). Y27632 was a potent PNK3 inhibitor, with a *K*_i_ value of 18 nM with respect to ATP and a *K*_i,app_ of 130 nM with respect to peptide substrate. The PKN3-PRL inhibitor was uncompetitive with respect to ATP and competitive with respect to peptide substrate (Figure 5B) with a *K*_i_ value of 5.4 μM with respect to peptide substrate, and an α*K*_i_ value of 1.3 μM with respect to ATP. This data further supports an ordered mechanism where ATP binding is followed by the binding of peptide substrate (Scheme 2). If substrate binding was random, PKN3-PRL would have been a non-competitive inhibitor with respect to ATP.

### Isoform selective PKN inhibitors

To our knowledge, this is the first report of Y27632 as a potent PKN3 inhibitor, while other reports have classified Y27632 as a PKN1/2 inhibitor with IC_50_ values in the submicromolar range [28,30]. The selectivity of Y27632 for PKN3 led us to screen multiple commercial kinase inhibitor libraries in an attempt to identify tool compounds that could be used to dissect PKN biology, as well as determine the feasibility of PKN isoform selective inhibitors. Representative screening ‘hits’ that included compounds with varying degrees of isoform selectivity were titrated, and the *K*_i_ values were determined in the absence of lipids (Table 3). All PKN isoforms were inhibited by PKC412, a staurosporine derivative, with *K*_i_ values in the subnanomolar range. Both PKN1 and PKN2 were inhibited by the structurally related bisindoylmaleimides, Ro 31-8220 and GF 109203X (PKC kinase inhibitors). However, GF 109203X exhibited at least 25-fold selectivity for PKN1/2 over PKN3, while Ro 31-8220 exhibited about a 9-fold selectivity for PKN1/2 over PKN3. Compound 6 (Cdk1/2 inhibitor III) was the most selective (18-fold) for PKN2 over PKN1 with only minimal selectivity over PKN3. Several potent and strikingly isoform-selective PKN3 inhibitors were identified (Table 4), including the Src kinase inhibitor PP1, the p38 MAPK (mitogen-activated protein kinase) inhibitor SB-202190, and the PKA (protein kinase A)/PKG (protein kinase G) inhibitor H-8. The results of our screen indicate that it is possible to obtain potent and selective small molecule inhibitors for PKN3 over PKN1/2, and that it may be possible to achieve inhibitor selectivity between PKN1 and PKN2.

**Table 3:** PKN inhibitors with differential isoform selectivity *K*_i_ values (±S.D., *n*=2) were determined using the off-chip mobility shift assay, and by fitting inhibition data to the equation for competitive inhibition, as described under the Experimental section.

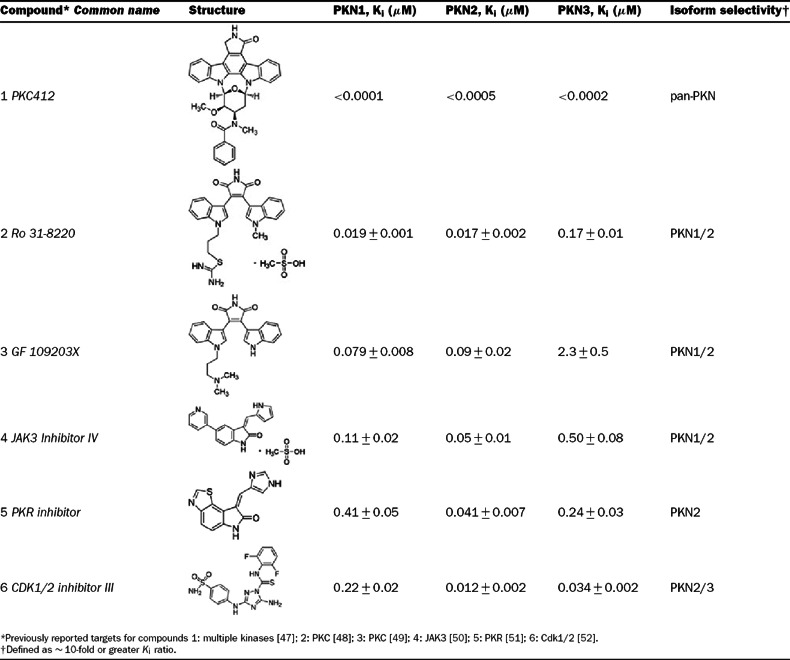

**Table 4:** PKN3 selective inhibitors *K*_i_ values ±S.D. (*n*=2) were determined using the off-chip mobility shift assay, and by fitting inhibition data to the equation for competitive inhibition, as described under the Experimental section.

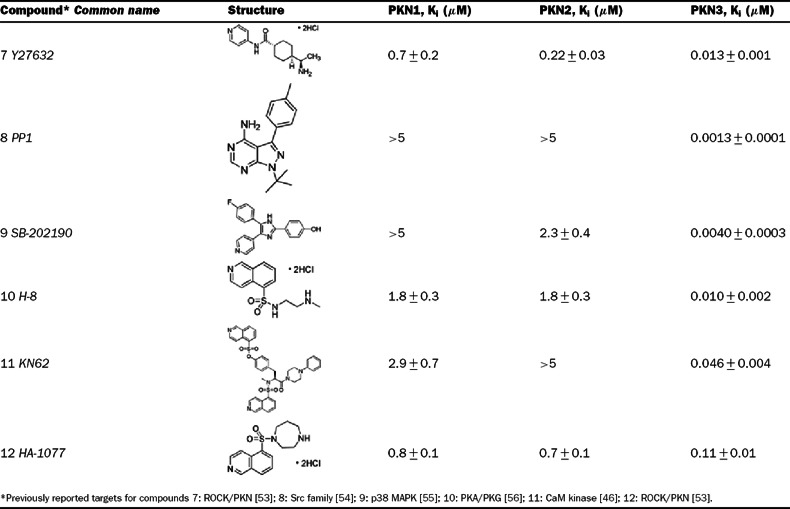

## DISCUSSION

Published works have highlighted the possible involvement of each of the three PKN isoforms in cancer, and has implicated each as a promising therapeutic target [22–26]. To effectively target this important family of kinases for pharmaceutical intervention, a greater understanding of their activity and regulation is required. We have expressed and purified full-length, human PKN1–3 from mammalian cells for kinetic evaluation, and shown these proteins to be phosphorylated at two critical points of regulation: the threonine in the activation loop, and the threonine/serine in the turn motif.

Our direct comparison of the effects of various signalling lipids on PKN activity resulted in striking differences on catalytic activity (Figure 2). PKN1 kinase activity was activated by all lipids tested, except IP3 (D-myo-inositol-1,3,5-triphosphate) and DAG (1,2-dioctanoyl-sn-glycerol), while PKN2 was only activated by PIP2 and arachidonic acid. We also showed, through bisubstrate kinetic analysis, that arachidonic acid increased the catalytic efficiency of each PKN isoform, mainly through an increase in *k*_cat_ for PKN1 and PKN2, and a decrease in *K*_M,Pep_ for PKN3. Similarly, a much larger relative stimulation of PKN1 over PKN3 by arachidonic acid was previously reported using a peptide substrate based on the PKCδ pseudosubstrate sequence [34]. The significance of specific isoform modulation by lipids, without structural insight, is unclear, and further studies are warranted. The differential sensitivities of these kinases to lipids may, however, provide insight into spatial regulation of their cellular signalling. PKN1 and PKN2 kinase activity in our biochemical studies was activated in the presence of PIP2 and arachidonic acid, major components of the cell membrane, while PKN3 was not, indicating that PKN1 and PKN2 may be primarily associated with the plasma membrane, while PKN3 may preferentially signal in other areas of the cell. Indeed, PKN1 has been shown to signal at the plasma membrane with RhoA [14], and the C2-domain of PKN2 has been shown to be required for its involvement in apical junction formation [20]. We have previously shown that activated PKN3 resides primarily in the nucleus [22], yet unactivated PKN3, based on turn motif phosphorylation, is found throughout the cell. We also showed that catalytic efficiency for PKN3 is significantly more favourable in the presence of low concentration of arachidonic acid. Experiments using radiolabelled arachidonic acid demonstrated that the nuclear membrane is highly labelled after cellular administration [39]. Since RhoC interaction with PKN3 may facilitate nucleocytoplasmic shuttling [22,40], one can hypothesize that after RhoC-mediated PKN3 translocation to the nucleus, arachidonic acid may interact with the kinase to increase the efficiency of substrate interaction and turnover. Both stimulatory concentrations of arachidonic acid for PKN1 and PKN2 (EC_50_ near 10 μM in biochemical experiments) appear to fall within the biologically relevant levels [41]. Interestingly, physiological PIP2 concentrations are considered to be constitutively high in many cell types, while PIP3 levels tend to rapidly rise during times of actin polymerization [42], a process in which PKN1 is implicated [17]. It is possible that rising levels of PIP3 (or other lipids not tested) could serve as a physiological switch to regulate PKN isoform signalling by relatively amplifying catalytic activity of PKN1 that, in contrast to PKN2 and PKN3, was strongly activated by PIP3. Whether lack of activation or partial inactivation of PKN2 and PKN3 by PIP3 or other lipids is a physiological response, and a marker of non-redundant functions between the PKN isoforms, remains to be investigated.

**Figure 6:**
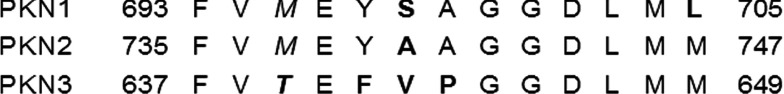
Multiple sequence alignment of the PKN protein kinase family in the kinase domain hinge region Protein sequences for PKN1–3 (UniProtKB Q16512, Q16513 and Q6P5Z2) were aligned using NCBI-BLAST [57]. The positions of the gatekeeper (italicized) and the rest of the hinge region correspond to those reported for other protein kinases [58–61]. Amino acids unique for each PKN isoform are shown in bold.

Using full-length recombinant human protein, the results of our kinetic study show that the PKN family of kinases follows an ordered Bi–Bi reaction mechanism, where ATP is the first substrate to bind, thus allowing subsequent peptide substrate binding and initiating the phosphotransfer steps. Since ATP binding is the initiating catalytic step, targeting the ATP binding site of PKN1–3 with small molecule inhibitors would likely be an effective route to block enzymatic function. A perhaps unexpected result of our study was that Y27632, a moderately potent PKN1/2 inhibitor, is a much more potent inhibitor of PKN3. In fact, multiple compounds displayed striking nanomolar potency and selectivity for PKN3 over PKN1/2 (Table 4), including the Src family inhibitor PP1, the p38 inhibitor SB-202190, and the CaM (calmodulin) kinase II inhibitor KN62. It is possible that the large selectivity margin seen with these compounds could be due to a difference in gatekeeper residues between PKN1/2, which contain a methionine, and PKN3, which contains a threonine (Figure 6). A proline in the hinge region of PKN3 may also be a site for achieving specificity, as this residue is an alanine in PKN1/2. Interestingly, the two reported PKN1/2 inhibitors, HA-1077 and Y27632, were much more potent against PKN3, displaying 7- and 54-fold selectivity for PKN3 over PKN1, respectively, which reclassifies these compounds as PKN3 inhibitors with PKN1 and PKN2 activity. With a low nanomolar *K*_i_ of 13 nM, Y27632 appears to be more potent against PKN3 than against its intended target of ROCK (Rho-associated kinase) [43]. This may have implications for interpreting the underlying mechanisms of action of Y27632 involving ROCK versus PKN3, for example, for inhibition of tumour metastasis in a PC3 prostate cancer xenograft model [44], a process in which PKN3 has been also implicated. The PKN inhibitors identified in Tables 3 and 4 could serve as useful chemical tools for dissecting PKN biology. Compounds 2, 4, 5 and 6 are broad-spectrum protein kinase inhibitors with distinct biochemical signatures from the panel profiling data [29,30,45]. When used collectively, these can be suitable, for example, to explore cellular PKN2 functions. Interestingly, several identified inhibitors (compounds 2–4) exhibited significant selectivity for

PKN1 and PKN2 over PKN3, consistent with a greater similarity of PKN1 and PKN2 in the hinge region (Figure 6). Compounds 7–12 could be extremely useful for studying PKN3 signalling, as these are narrow-spectrum inhibitors [29,30,46]. Compounds 8–10 produced single-digit nanomolar PKN3 *K*_i_ values. Of these, compound 8 (PP1) was the most potent for PKN3 and demonstrated a surprisingly large margins (>5000-fold) of isoform selectivity. PP1 inhibited relatively few kinases in a panel profiling of over 70 protein kinases at University of Dundee [29]. The IC_50_ values for submicromolar ‘hits’ of PP1 were reported as follows: RIP2 (0.026 μM), Lck (0.040 μM), Src (0.053 μM), CK1δ (0.17 μM) and CSK (0.64 μM). Consistent with our results, PKN2 was poorly inhibited by PP1 (84% remaining activity at 1 μM) and moderately inhibited by Y27632 (IC_50_=600 nM) in the panel profiling studies [29,30], but PKN1 and PKN3 were not part of the panel. Taken together, our results indicate that, while the PKN family may share a common reaction mechanism, intrinsic catalytic properties of the PKN isoforms may prove useful in designing PKN-targeted agents. The identified structurally diverse small-molecule inhibitors with distinct biochemical signatures and various margins of isoform selectivity could be used as probes to discriminate cell functions of the members of this family.

## Online data

Supplementary data
