# Supplementary material for: Enzyme kinetics and distinct modulation of the protein kinase N family of kinases by lipid activators and small molecule inhibitors
Source: Biosci Rep. 2014 Mar 18;34(2):e00097. doi: 10.1042/BSR20140010 (PMC3958129; doi:10.1042/BSR20140010)
Supplement: Supplementary data [file bsr034e097add.pdf]

Biosci. Rep. (2014) / **34** / art:e00097 / doi 10.1042/BSR20140010

Bioscience Reports

www.bioscirep.org

Bioscience Reports

SUPPLEMENTARY DATA

Enzyme kinetics and distinct modulation of the protein kinase N family of kinases by lipid activators and small molecule inhibitors

Matthew D. FALK*, Wei LIU†, Ben BOLAÑOS†, Keziban UNSAL-KACMAZ‡, Anke KLIPPEL‡[1], Stephan GRANT*, Alexei BROOUN† and Sergei TIMOFEEVSKI*[2]

*Pfizer Oncology Research Unit, Pfizer Inc., San Diego, CA 92121, U.S.A.
†Pfizer Worldwide Medicinal Chemistry, Pfizer Inc., San Diego, CA 92121, U.S.A.
‡Pfizer Oncology Research Unit, Pfizer Inc., Pearl River, NY 10965, U.S.A.

Supplementary Figure S1 is on the following page.

1 Present address: Celgene Corporation, Summit, NJ 70901, U.S.A.
2 To whom correspondence should be addressed (email sergei.timofeevski@pfizer.com).

© 2014 The Author(s) This is an Open Access article distributed under the terms of the Creative Commons Attribution Licence (CC-BY) (http://creativecommons.org/licenses/by/3.0/) which permits unrestricted use, distribution and reproduction in any medium, provided the original work is properly cited.

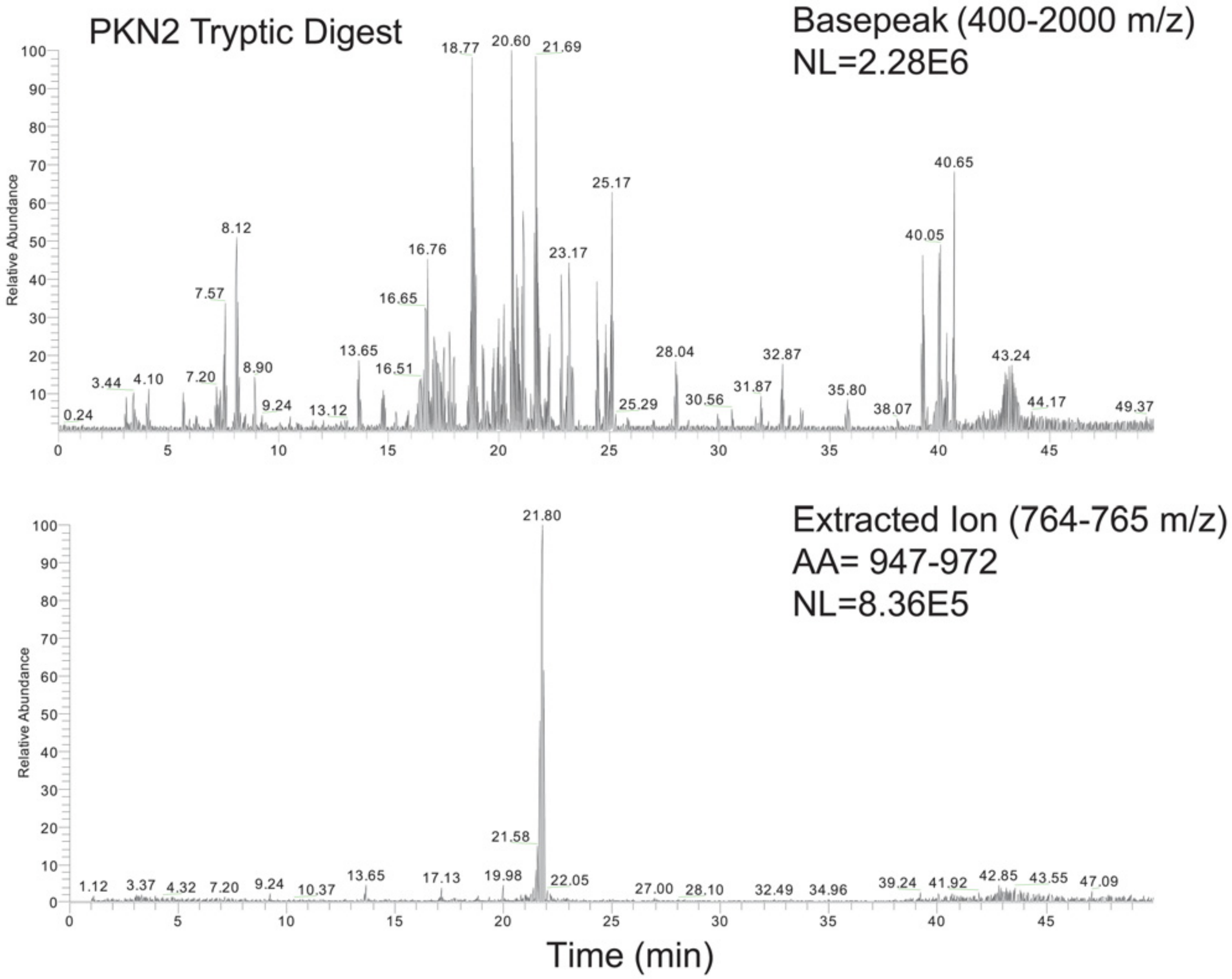

Figure S1 **(Top) LC-MS basepeak chromatograph for PKN2 tryptic digest; (bottom) extracted MS ion chromatograph for phosphorylated turn-motif peptide, amino acids 947–972**

Received 14 January 2014; accepted 29 January 2014

Published as Immediate Publication 29 January 2014, doi 10.1042/BSR20140010

© 2014 The Author(s) This is an Open Access article distributed under the terms of the Creative Commons Attribution Licence (CC-BY) (http://creativecommons.org/licenses/by/3.0/) which permits unrestricted use, distribution and reproduction in any medium, provided the original work is properly cited.
